# Etanercept does not impair healing in rat models of tendon or metaphyseal bone injury

**DOI:** 10.3109/17453674.2012.693018

**Published:** 2012-06-04

**Authors:** Olof Sandberg, Pernilla Eliasson, Therese Andersson, Fredik Agholme, Per Aspenberg

**Affiliations:** Orthopedics Division, Department of Clinical and Experimental Medicine, Linköping University, SE-581 85 Linköping, Sweden; Correspondence: olof.sandberg@liu.se

## Abstract

**Background and purpose:**

Should blockade of TNF-α be avoided after orthopedic surgery? Healing of injuries in soft tissues and bone starts with a brief inflammatory phase. Modulation of inflammatory signaling might therefore interfere with healing. For example, Cox inhibitors impair healing in animal models of tendon, ligament, and bone injury, as well as in fracture patients. TNF-α is expressed locally at increased levels during early healing of these tissues. We therefore investigated whether blocking of TNF-α with etanercept influences the healing process in established rat models of injury of tendons and metaphyseal bone.

**Methods:**

Rats were injected with etanercept, 3.5 mg/kg 3 times a week. Healing of transected Achilles tendons and bone healing around screws implanted in the tibial metaphysis were estimated by mechanical testing. Tendons were allowed to heal either with or without mechanical loading. Ectopic bone induction following intramuscular BMP-2 implants has previously been shown to be stimulated by etanercept in rodents. This was now tested as a positive control.

**Results:**

Tendon peak force after 10 days was not significantly influenced by etanercept. Changes exceeding 29% could be excluded with 95% confidence. Likewise, screw pull-out force was not significantly influenced. More than 25% decrease or 18% increase could be excluded with 95% confidence. However, etanercept treatment increased the amount of bone induced by intramuscular BMP-2 implants, as estimated by blind histological scoring.

**Interpretation:**

Etanercept does not appear to impair tendon or metaphyseal bone healing to any substantial degree.

Inflammation is an important part of early healing of both bone and soft tissues. Several inflammatory mediators are known to be expressed and to influence tissue healing. Non-steroidal anti-inflammatory drugs (NSAIDs) inhibit cyclooxygenases and direct eicosanoid synthesis from prostaglandins towards leukotrienes (Cottrell and O’Connor 2009). It has been known for decades that these drugs inhibit fracture repair in animal models; later, it was shown that they also inhibit tendon and ligament healing (Elder et al. 2001, Virchenko et al. 2004). It has also been shown that NSAIDs increase the risk of healing complications in patients with high-energy fractures (Burd et al. 2003). Clinicians now take this into account and avoid using NSAIDs in cases where bone healing might become problematic. Corticosteroids inhibit fracture healing in animal experiments, possibly through its anti-inflammatory action (Waters et al. 2000, Doyon et al. 2010). Other drugs that modulate inflammatory mediators, such as modulators of tumor necrosis factor α (TNF-α) signaling, have not been studied much in the fracture healing context.

TNF-α is expressed during fracture healing (Kon et al. 2001). Also, we have noted strong TNF-α expression during early tendon healing, and that mechanical loading can reduce this expression (Eliasson et al. 2009a). There are few data available on how modulation of TNF-α signaling influences repair. Systemic treatment with TNF-α was found to reduce the amount of cartilage formed in rib fractures in rats (Hashimoto et al. 1989), and antagonizing the effect of TNF-α with etanercept increased bone formation in bone morphogenic protein-2 (BMP-2) implants in mice (Eguchi et al. 2010). Mice unable to express TNF-α receptor p55/p75 have delayed cartilage resorption during endochondral fracture healing (Gerstenfeld et al. 2003). TNF-α signaling appears to be required for normal membranous ossification, as this type of bone formation is reduced in receptor knockout mice (Gerstenfeld et al. 2001). Recently, it was suggested that TNF-α was crucial for recruiting muscle-derived stem cells (MSCs) into fracture hematomas in a mouse delayed-union model (Glass et al. 2011). The same article also briefly described a positive effect on mouse tibial fracture healing of local injections of TNF-α on day 0 and 1 after fracture, as measured on day 28. Although studies with receptor knockouts or local injection of TNF-α can give mechanistic insights, they may not be ideal for predicting the effects of etanercept treatment.

Clinically, fractures in metaphyseal bone, which heal mainly by membranous endosteal bone formation, are much more common than diaphyseal fractures, which heal partly by endochondral repair. Ruptures in soft collagenous tissues are also common. Because the in vivo functions of TNF-α appear complex, even a detailed knowledge of its effects at the cellular level is probably insufficient to be able to accurately predict the consequences of changing TNF-α signaling during a healing process. We therefore studied the effects of etanercept on tendon healing and endosteal membranous ossification in vivo using validated rat models, where the results of both of these processes can be tested mechanically.

Short, early NSAID treatment can impair healing. It appears that early inflammation may be necessary to trigger healing, while late inflammation could be deleterious (Virchenko et al. 2004). We therefore tested etanercept treatment both in the early phase and later in separate groups. Because etanercept has been shown to increase bone formation in intramuscular BMP-2 implants (Eguchi et al. 2010), we used that model as a form of positive control to demonstrate that the etanercept regime was adequate.

## Material and methods

### Experimental overview

We used 110 rats in 3 different experiments, evaluating the effects of etanercept treatment on healing of transected Achilles tendons, on healing of metaphyseal bone injury due to screw insertion, and on intramuscular bone formation in response to BMP-containing collagen implants.

### Animals, anesthesia, and dosing

The experiments were approved by the regional ethics committee for animal experiments and we followed the institutional guidelines for care and treatment of experimental animals. The animals were randomly allocated to treatment groups with 10 in each (10-week-old Sprague-Dawley rats (Taconic, Lille Skensved, Denmark)). They shared cages in groups of 2 to 4, with 12-h light/dark cycles, a room temperature of 21˚C, and free access to food and water. They were killed using CO_2_ gas.

During surgery, all animals were anesthetized with 5% isoﬂurane gas, prior to which they were given 25 mg/kg tetracycline as antibiotic. As analgesic, 0.045 mg/kg buprenorphine was given before surgery and after 12 and 24 h. The surgery was performed under aseptic conditions as previously described (Wermelin et al. 2008, Andersson et al. 2009). Surgeons and evaluators were blinded as to treatment.

The dosage of etanercept (Enbrel; Wyeth) was always 3.5 mg per kg body weight. Rats receiving etanercept on day 0 were given it 1 h before the operation.

### Tendon transection experiment

The effect of etanercept on Achilles tendon healing was studied in 40 female rats. Half of the rats received botulinum toxin injections (Botox; Allergan, Irvine, CA) into the calf muscles of their right hind leg 5 days before surgery, in order to study healing under different loading conditions. 3 injections of 1 U each (total injected volume 0.06 mL) were administered. All animals developed an obvious limp that remained for the duration of the experiment.

During the surgical procedure, the right Achilles tendon was exposed and a 3-mm segment was removed. The plantaris tendon was removed entirely and the Achilles tendon left unsutured (Eliasson et al. 2009b). Etanercept was given on days 0, 2, 5, and 8, or not at all. The animals were killed on day 10, and the strength of the healing tendon was measured.

### Metaphyseal screw experiment

The effect of etanercept on metaphyseal bone healing was assessed in 40 male rats by mechanical pull-out testing of an implanted screw. An 8- to 9-mm incision was made along the left proximal tibia. A 1.4-mm hole was then drilled, approximately 3 mm from the physis. Thereafter, a stainless steel screw, 1.6 mm in diameter, was inserted as previously described (Wermelin et al. 2008). Etanercept was administered on days 0 and 2, days 5, 8, and 11, days 0, 2, 5, 8, and 11, or not at all. The animals were killed and pull-out force, stiffness, and energy measured on day 14.

### Intramuscular implant experiment

As an indicator of the biological effect of the etanercept dose chosen, 30 male rats received a collagen carrier with 30 µg rh-BMP-2 inserted into an abdominal muscle pouch created between the inner and outer oblique muscle layers, just lateral to the rectus sheath. A 3 × 2.5 × 5 mm collagen sponge (Helistat; Colla-Tec Inc., Plainsboro, NJ) was soaked with 10 µL 0.2 mM acetic acid containing 30 µg of rh-BMP 2 (GenScript, Piscataway, NJ) and inserted into the pouches (Aspenberg and Turek 1996). Etanercept was given on day 0 and 2, on days 0, 2, 5, 8, and 11, or not at all. The animals were killed on day 14. Bone formation in the implants was assessed by radiology and histology.

### Mechanical evaluation

Mechanical evaluation was performed using a computerized materials testing machine (100R; DDL Inc., Eden Prarie, MN). The crosshead speed was 0.1 mm/s. Samples were kept moist and evaluated within an hour after harvest. Peak force (N) was considered the primary effect variable. The energy (Nmm) absorbed until the force reached its peak value was measured for the tendons. For screw pull-out testing, the energy absorbed until the force dropped to 90% of the peak value was measured instead. Stiffness (N/mm) was recorded for both. Methods for tissue mounting in the testing machine have been described (Virchenko et al. 2004, Wermelin et al. 2008).

### Radiographic analysis

The collagen BMP implants could be identified by transillumination of the abdominal wall. Thereafter, the pieces were trimmed down, fixed in buffered formalin, and radiographed (MX-20; Faxitron X-ray Corp., Chicago, IL). The pictures were analyzed by image processing using 2 arbitrarily chosen thresholding levels. These levels were the same for all samples. A lower threshold was used to estimate the total area of the ossicle, and a higher threshold to estimate mineral content (ImageJ Software; NIH, Bethesda, MD).

### Histology

Following radiographic evaluation, the collagen BMP implants were decalcified, paraffin embedded, and prepared for histology with hematoxylin and eosin staining. The slides were assessed by blinded qualitative scoring and given a value from 0 to 4 for each of 4 variables, namely the amounts of bone, marrow, cartilage, and remaining collagen carrier.

### Statistics

We tested tendon mechanics with two-way ANOVA, using loading status (Botox or no Botox) and drug treatment as independent factors. Peak force served as the predetermined primary variable. 95% confidence intervals (CIs) for the treatment effects were calculated separately for loaded tendons and unloaded tendons.

Screw fixation was tested by one-way ANOVA. Peak pull-out force served as the predetermined primary variable. For calculation of the CI for treatment effects, controls were compared with all etanercept groups combined.

The area of the induced ectopic ossicle, and an estimate of its mineral density, were tested by one-way ANOVA. Histological scores were tested by Kruskall-Wallis test, followed by Mann-Whitney test for intergroup comparisons. We used PASW 18.0 software.

## Results

### Tendon healing

**Figure 1.**
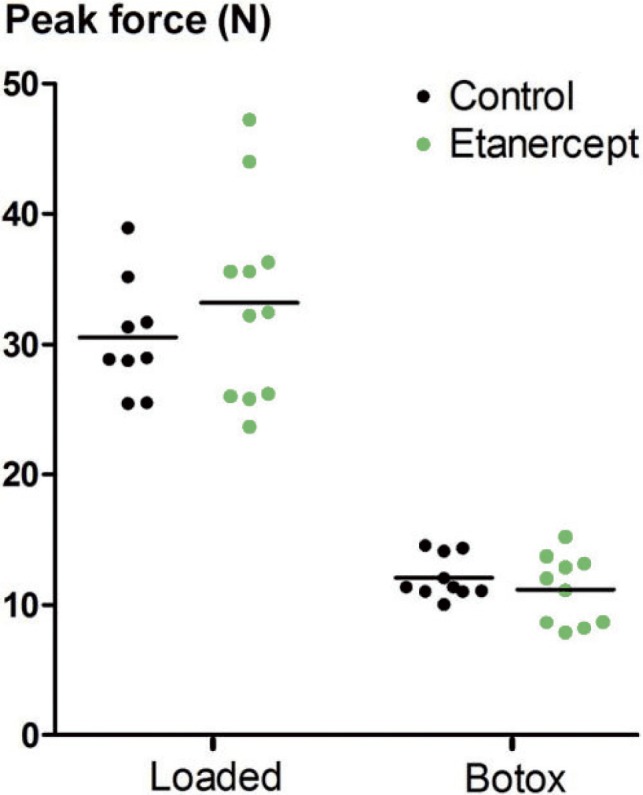
Peak force (N) at tensile testing of transected tendons that were allowed to heal either with voluntary loading or with muscle paralysis induced by Botox.

Load protection by use of Botox reduced the peak force by almost two-thirds in both control rats and etanercept-treated rats, but there was no statistically significant effect of etanercept (Figure 1). For the loaded tendons, the CI for the treatment effect of etanercept ranged from a decrease in force of 11% to an increase of 29%. For the load-protected tendons, the range was between a decrease of 25% and an increase of 9%. Similar results were seen for stiffness and energy uptake (Table 1).

**Table 1.** 95% confidence limits for the difference between means for mechanical properties of transected tendons (etanercept minus control). Values are expressed as percent of control (percent change from etanercept). The tendons were allowed to heal with voluntary loading or with muscle paralysis induced by Botox

	Lower	Mean	Upper
Loaded			
Peak force	–11	9	29
Stiffness	–21	5	31
Energy	–14	16	45
Botox			
Peak force	–25	-8	9
Stiffness	–27	-9	9
Energy	–23	-3	16

### Bone healing

**Figure 2.**
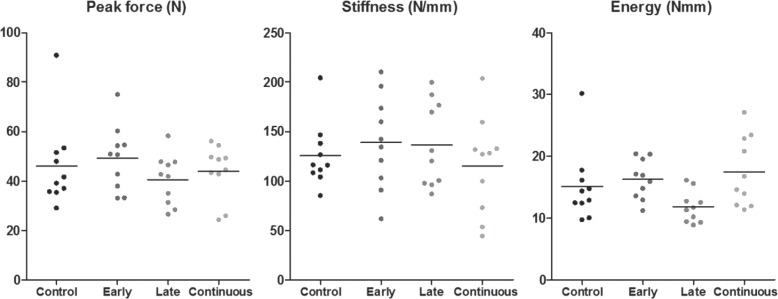
Data from pull-out testing of screws 14 days after insertion in the proximal tibial metaphysis. Etanercept was given early, late, or continuously over the 14-day period.

Screw peak pull-out force was not statistically significantly influenced by any of the etanercept treatment regimes (Figure 2). The CI for the treatment effect of etanercept ranged from a decrease in force of 25% to an increase of 18%. Similar results were obtained for stiffness and energy uptake (Table 2).

**Table 2.** 95% confidence limits for the difference between means for screw fixation (the etanercept groups combined minus controls). Values are expressed as percent of control (percent change from etanercept)

	Lower	Mean	Upper
Peak force, N	–25	–3	18
Stiffness, N/mm	–22	4	29
Energy, Nmm	–24	1	25

### Ectopic bone formation

Histology showed new-formed ossicles in all cases. They consisted of a shell of woven bone surrounding a marrow cavity with hematopoesis and fat cells. Within the woven bone, isolated chondrocyte-like cells were sometimes seen, but these were rare. Continuous areas of cartilage matrix were never seen.

**Figure 3.**
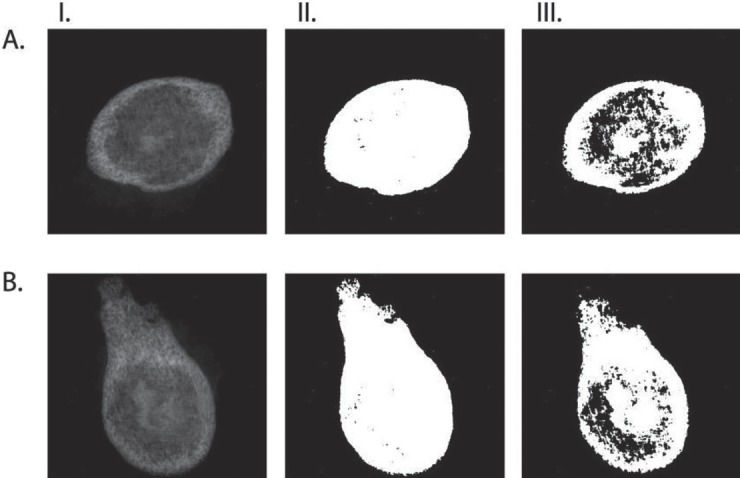
Ossicles induced by BMP-2 in a collagen scaffold. Row A: control. Row B: continuously etanercept-treated. Column I: raw image. Column II: low threshold (ossicle size). Column III: high threshold (estimate of bone density).

**Figure 4.**
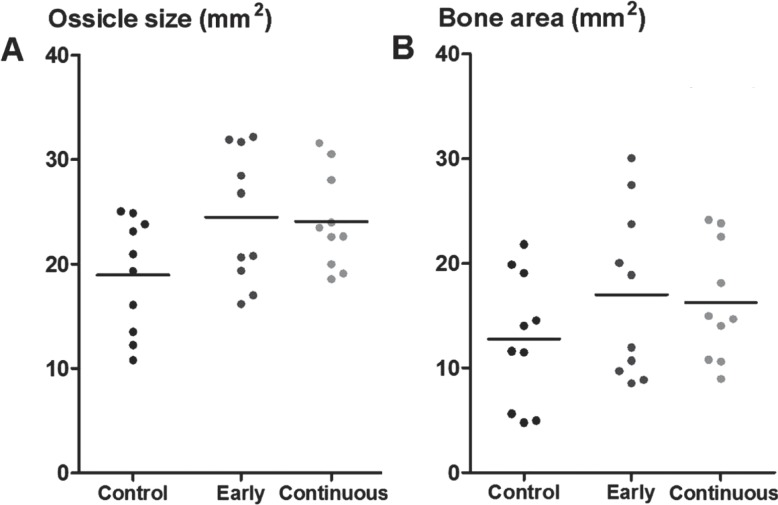
Radiographic data from rh-BMP 2 implants, with two different gray-scale thresholds for blind examination. Ossicle size (A) was measured with a low threshold, and “bone area” (B) with a higher threshold, giving a visual impression of being related to bone density. For comparison, both estimates are presented in mm2. Ossicle size was larger with etanercept treatment (pooled; p = 0.02).

The total area of the formed ossicle on the radiographs was not significantly different in the 3 treatment groups, as shown by one-way ANOVA (p = 0.06) (Figure 3). Therefore, to increase power, the etanercept groups were pooled and compared with the controls by a t-test, which showed a larger total area with etanercept treatment, compared to the controls (p = 0.02) (Figure 4 and Table 3). However, no statistically significant effect was seen on the amount of bone within the ossicle, as estimated by radiodensity with a higher threshold (Figure 3 and Table 3).

**Table 3.** 95% confidence limits for the difference between means for radiographic data describing ossicles induced by BMP-2 (both the etanercept groups combined minus controls). Values are expressed as percent of control (percent change from etanercept)

	Lower	Mean	Upper
Ossicle size	5	28	51
Bone area	–11	30	71

Qualitative blind scoring indicated effects on the amount or density of bone (p = 0.02) and the amount of remaining collagen (p = 0.02) (Table 4). Post hoc testing showed that the amount of bone was higher in the etanercept groups than in the controls (p = 0.008 for continuous treatment and p = 0.06 for early treatment). The amount of remaining collagen carrier was reduced in the etanercept groups (p = 0.2 for continuous treatment and p = 0.004 for early treatment). Scores for cartilage and marrow were similar in the different groups.

**Table 4.** Histological scoring of endochondral ossification: median (min–max). The scale goes from 0 (no prevalence) to 3 (high prevalence). The p-values are from Kruskall-Wallis test

	Control	Early	Continuous	Combined	p-value
Bone	1 (1–2)	2 (1–3)	3 (1–3)	2 (1–3)	0.02
Marrow	3 (2–3)	3 (2–3)	2 (1–3)	2 (1–3)	0.1
Cartilage	1 (0–2)	1 (0–2)	1 (0–2)	1 (0–2)	0.9
Collagen scaffold	2 (1–2)	1 (0–1)	1 (0–2)	1 (0–2)	0.02

## Discussion

With reasonable confidence, from our results we can exclude the possibility that etanercept has moderate to strong negative effects on metaphyseal bone and tendon healing. Previous tendon healing experiments with similar protocols and of comparable power showed that NSAID treatment had statistically significantly negative effects (Virchenko et al. 2004), suggesting that etanercept is less detrimental, although no direct comparison has been made.

We believe that the etanercept was given at a relevant dose, for two reasons. Firstly, we used a dose that has previously been shown to clearly modify disease processes in several rat models (Boettger et al. 2008, Zanella et al. 2008, Hsu and Chang 2010). Secondly, as a positive control in the present experiment, etanercept increased bone formation in intramuscular BMP 2 implants. However, we cannot definitively exclude the possibility that the doses tested were irrelevant.

Results for tendon healing in the rat model used might also be relevant for ligaments. For example, NSAIDs have similar effects on the healing of rat tendons and ligaments. To our knowledge, the effects of TNF-α signaling on the healing of these tissues have not been described, possibly because they appear to be weak.

In a study with intramuscular BMP-2 implants in mice, etanercept was found to increase the amount of ectopic bone formed (Eguchi et al. 2010). In that study, no cartilage was seen, but the authors postulated that cartilage formation had preceded bone formation. We saw isolated chondrocyte-like cells, but no cartilage, and the bone structure resembled fetal, woven bone. This suggests that the bone had formed directly without any endochondral process. It has been shown in similar models that direct bone formation predominates if high doses of BMP are given (Murata et al. 1999). Thus, our results may not be valid for endochondral bone formation during fracture repair. Moreover, metaphyseal bone healing and ectopic bone formation probably originate from different cells. In metaphyseal marrow, MSCs and osteoblast progenitors may be abundant, whereas ectopic bone formation may be dependent on recruitment of MSCs from surrounding muscle or from the circulation.

The pathophysiological role of TNF-α signaling during healing is unclear, although it is known that TNF-α expression increases soon after injury (Kon et al. 2001). Deletion of TNF-α receptors in mice impairs bone healing, both in shaft fractures and after marrow ablation, which indicates that TNF-α signaling is required for good healing (Gerstenfeld et al. 2001). It appears that TNF-α aids in MSC recruitment—and also in the transition from cartilage to bone via chondrocyte apoptosis and osteoclast recruitment (Gerstenfeld et al. 2003). On the other hand, local injection of TNF-α reduced the amount of cartilaginous callus in rat rib fractures, indicating that high levels of TNF-α are detrimental (Hashimoto et al. 1989).

The interpretation of our findings is limited by moderately wide confidence intervals, the use of only single time points, and especially by our incomplete knowledge of how results in rats would relate to humans.

To conclude, our observations do not support the idea that blocked TNF-α signaling poses a serious risk to healing of metaphyseal fractures or tendon injuries.
